# Dysregulated cytokine and oxidative response in hyper-glycolytic monocytes in obesity

**DOI:** 10.3389/fimmu.2024.1416543

**Published:** 2024-07-10

**Authors:** Veselina Radushev, Isabel Karkossa, Janina Berg, Martin von Bergen, Beatrice Engelmann, Ulrike Rolle-Kampczyk, Matthias Blüher, Ulf Wagner, Kristin Schubert, Manuela Rossol

**Affiliations:** ^1^ Division of Rheumatology, Department of Endocrinology, Nephrology, Rheumatology, Leipzig University, Leipzig, Germany; ^2^ Department of Molecular Toxicology, Helmholtz Centre for Environmental Research GmbH, Leipzig, Germany; ^3^ Molecular Immunology, Faculty of Health Sciences, BTU Cottbus-Senftenberg, Senftenberg, Germany; ^4^ Institute for Biochemistry, Faculty of Life Sciences, Leipzig University, Leipzig, Germany; ^5^ German Centre for Integrative Biodiversity Research (iDiv) Halle-Jena-Leipzig, Leipzig, Germany; ^6^ Helmholtz Institute for Metabolic, Obesity and Vascular Research (HI-MAG) of the Helmholtz Zentrum München at the University of Leipzig and University Hospital Leipzig, Leipzig, Germany; ^7^ Faculty of Environment and Natural Sciences, BTU Cottbus-Senftenberg, Senftenberg, Germany

**Keywords:** monocytes, immunometabolism, respiratory burst, obesity, IL-8

## Abstract

**Introduction:**

Obesity is associated with a plethora of health complications, including increased susceptibility to infections or decreased vaccine efficacy, partly due to dysregulated immune responses. Monocytes play a crucial role in innate immunity, yet their functional alterations in obesity remain poorly understood.

**Methods:**

Here, we employed proteomic and metabolomic analyses to investigate monocyte characteristics in individuals with overweight, obesity, impaired glucose tolerance (IGT), and type 2 diabetes (T2D), compared to lean donors.

**Results and discussion:**

Our results revealed distinct molecular signatures in monocytes from individuals with obesity, with significant alterations in pathways related to metabolism, cellular migration, and phagocytosis. Moreover, LPS-induced activation of monocytes unveiled heightened metabolic reprogramming towards glycolysis in subjects with obesity accompanied by dysregulated cytokine responses and elevated oxidative stress. Additionally, monocytes from donors with obesity exhibited increased lipid droplet accumulation. These findings shed light on the immunometabolic dysregulation underlying obesity-associated immune dysfunction, highlighting potential targets for therapeutic intervention.

## Introduction

Obesity is characterized by the excessive accumulation of body fat and the increased risk of developing impaired glucose tolerance (IGT), type 2 diabetes (T2D), dyslipidemia, myocardial infarction, and some types of cancer (1). The accumulation of visceral adipose tissue macrophages is also a hallmark of obesity, and these cells secrete pro-inflammatory cytokines like TNF, IL-6, and IL-1ß, driving systemic, chronic, low-grade inflammation (2–4). Similar to chronic inflammation in aging (inflammaging), obesity-associated inflammation leads to premature aging of the immune system (5–7), contributing to the increased risk of obese people getting bacterial, viral, and fungal infections or having a more severe outcome (8–10), and to the decreased vaccine effectiveness in obese people (10, 11), as seen in the COVID-19 pandemic (12, 13).

Peripheral blood monocytes are part of the innate immune system in the first line of defense against pathogens. They are precursors of tissue macrophages, and monocyte migration contributes to macrophage accumulation in adipose tissue (2, 3, 14). Adipose tissue macrophages in obesity are well characterized (15–17). Little is known, however, about monocyte functions in human obesity. Obesity is associated with a myeloid lineage bias, an increased number of blood monocytes, and dysregulated monocyte subpopulations, partially induced by low-grade inflammation (2, 18–20). The expansion of myeloid lineages is at the expense of the lymphoid compartment in obesity, leading to enhanced metastatic progression (20). The expression of TLR4 and the inflammatory response to lipopolysaccharide (LPS) was found to be increased in classical monocytes of people with obesity (21), and the expression of chemokine receptors and the migratory capacity of monocytes was enhanced in people with obesity (21, 22). Nevertheless, the underlying molecular mechanisms have not been characterized yet.

Hence, the aim of this study was to analyze the immunometabolic and functional status of monocytes from people with obesity. Monocytes were characterized using proteomics, metabolomics, and analysis of immunological functions. We found that monocytes of people with obesity are hyper-glycolytic and have a dysregulated cytokine and oxidative response to bacterial lipopolysaccharide.

## Results

### Monocytes from people with obesity show a distinct proteomic profile

To characterize the molecular signature of circulating classical monocytes (isolated by negative selection) from donors with overweight, obesity, obesity with impaired glucose tolerance (IGT) and obesity with type 2 diabetes (T2D) compared to lean donors, LC-MS/MS-based global proteomics was used. Monocytes were analysed *ex vivo*, which resulted in 2350 quantified proteins in at least 3 of 4 donors.

**Figure 1:**
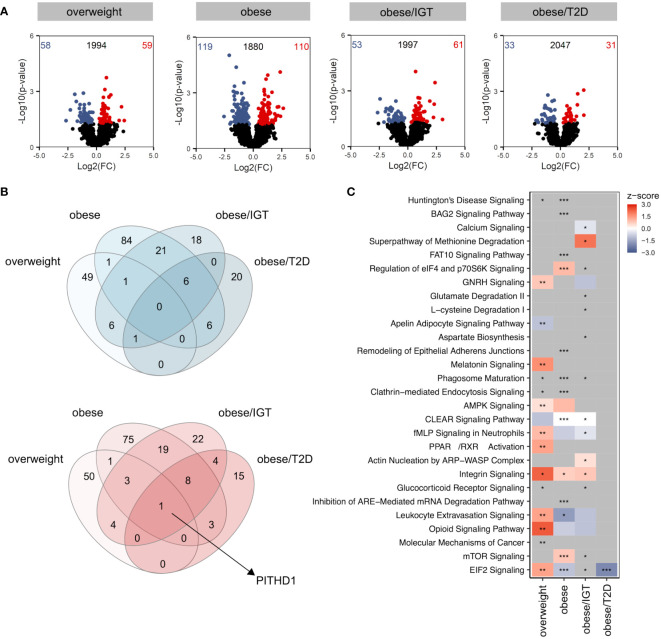
Proteomic profile of monocytes from donors with overweight, obesity, obesity/IGT and obesity/T2D compared to monocytes from lean donors *ex vivo*. **(A)** Volcano plot depicting the Log2(FC) vs. -Log10(p-value), highlighting the numbers of significantly up- (Log2(FC) > 0) or downregulated (Log2(FC) < 0) proteins (p. ≤ 0.05) from monocytes *ex vivo* from donors with overweight, obesity, obesity/IGT and obesity/T2D compared to monocytes from lean donors (*n* = 4). Protein names of proteins ≥ 95% percentile of the p-value are labeled. **(B)** Overlap of significantly up- (Log2(FC) > 0; red) or downregulated (Log2(FC) < 0; blue) proteins (p. ≤ 0.05). **(C)** For each comparison, the top 10 significantly (adjusted p-value ≤ 0.05) enriched IPA pathways for each comparison were extracted, resulting in 24 significantly altered pathways. Shown are z-scores reflecting the direction of the regulation (red: upregulation, blue: downregulation, white: no direction; grey: no z-score was calculated) and asterisks indicating the significance of enrichment: * adjusted p-value ≤ 0.05, ** adjusted p-value ≤ 0.01, *** adjusted p-value ≤ 0.001.

Compared to lean donors, the most significant differences were observed in monocytes from people with obesity (**Figure 1A**), with only a slight overlap between the different groups when comparing up (red) and down (blue) regulated proteins (**Figure 1B**). The only protein significantly upregulated in all four groups was PITH containing-protein 1 (PITH1), which was shown to upregulate RUNX1 expression (23), a transcription factor regulating cytokines.

Analysing significantly altered core pathways using Ingenuity Pathway Analysis (IPA^®^) considering all proteins with a p-value ≤ 0.05, a distinct pattern was observable for the four groups compared to monocytes from lean donors (**Figure 1C**, **Additional File Table A3**). In monocytes from overweight donors, pathways related to migration (Actin nucleation by ARP-WASP Complex, Integrin Signalling, Leukocyte Extravasation Signalling) were activated (**Figure 1C**), which was not as clearly observed for monocytes from obese donors. All proteins involved that are covered in IPA’s chemotaxis pathway are shown in **Supplementary Figure 1** with different regulations in the four donor groups (**Supplementary Figure 1**). While monocytes from donors with obesity/T2D showed only little alterations on the pathway level, monocytes from donors with obesity with and without IGT exhibited changes in pathways related to phagocytosis, endocytosis or cellular metabolism (**Figure 1C**).

In summary, the proteomic analysis of monocytes from people with obesity and comorbidities unravelled a distinct molecular profile *ex vivo*.

### LPS-activation of monocytes from people with obesity induced a dysregulated metabolic and immune response compared to lean controls

**Figure 2:**
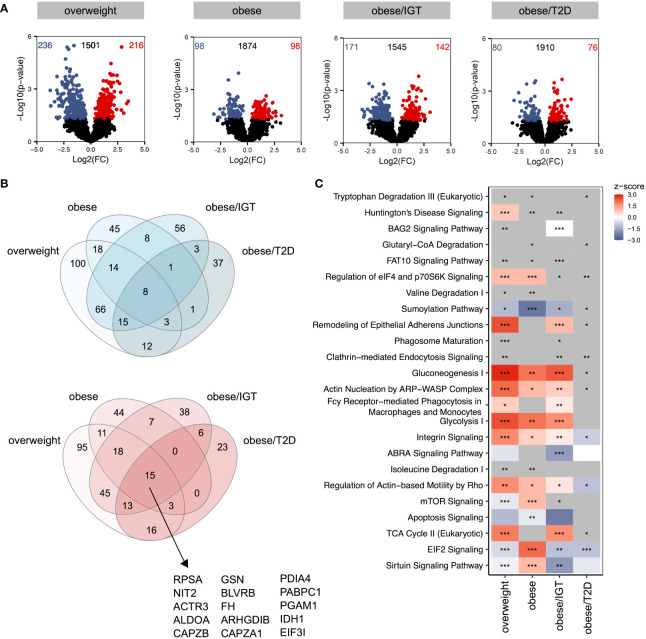
Proteomic profile of LPS-stimulated monocytes from donors with overweight, obesity, obesity/IGT and obesity/T2D compared to monocytes from lean donors. Monocytes were stimulated with 100ng/ml LPS for 3h (n=4). **(A)** Volcano plot depicting the Log2(FC) vs. -Log10(p-value), highlighting the numbers of significantly up- (Log2(FC) > 0) or downregulated (Log2(FC) < 0) proteins (p. ≤ 0.05) compared to monocytes from lean donors (*n* = 4). Protein names of proteins ≥ 95% percentile of the p-value are labeled. **(B)** Overlap of significantly up- (Log2(FC) > 0; red) or downregulated (Log2(FC) < 0; blue) proteins (p. ≤ 0.05). **(C)** For each comparison, the top 10 significantly (adjusted p-value ≤ 0.05) enriched IPA pathways for each comparison were extracted, resulting in 24 significantly altered pathways. Shown are z-scores reflecting the direction of the regulation (red: upregulation, blue: downregulation, white: no direction; grey: no z-score was calculated) and asterisks indicating the significance of enrichment: * adjusted p-value ≤ 0.05, ** adjusted p-value ≤ 0.01, *** adjusted p-value ≤ 0.001.

Considering that donors with obesity and associated comorbidities are more susceptible to bacterial, viral, and fungal infections, we next traced the molecular consequences of endotoxin-induced activation of classical monocytes from the same donor cohort using lipopolysaccharide (LPS). Log2(FC)s were calculated against monocytes from lean donors stimulated with LPS. Compared to the analysis of monocytes *ex vivo*, LPS-activated monocytes showed more significantly altered proteins in the four groups relative to monocytes from lean donors (**Figure 2A**) with more overlap between up- (red) and downregulated (blue) proteins (**Figure 2B**). The proteins that were upregulated in all four groups were mainly enzymes involved in cell metabolism, e.g. aldolase A (ALDOA), fumarase (FH), phosphoglycerate mutase 1 (PGAM1) or isocitrate dehydrogenase 1 (IDH1) (**Figure 2B**, **Supplementary Figure 2**). Interestingly, the protein disulfide isomerase family A member 4 (PDIA4) was also jointly upregulated in the 4 donor groups, which is an endoplasmatic stress protein modulating the immune response and insulin resistance in skeletal muscle (24). The analysis of affected pathways revealed that especially metabolic pathways like glycolysis, glyconeogenesis and TCA cycle were regulated in LPS-activated monocytes in people with obesity and comorbidities compared to lean donors (**Figure 2C**). As observed in monocytes *ex vivo*, pathways related to cellular migration and phagocytosis were also activated in monocytes from donors with obesity stimulated with LPS (**Figure 2C**).

Taken together, LPS-stimulation upregulated pathways involved in metabolism as well as phagocytosis and migration.

### Monocytes of people with obesity have a hyper-glycolytic phenotype

**Figure 3:**
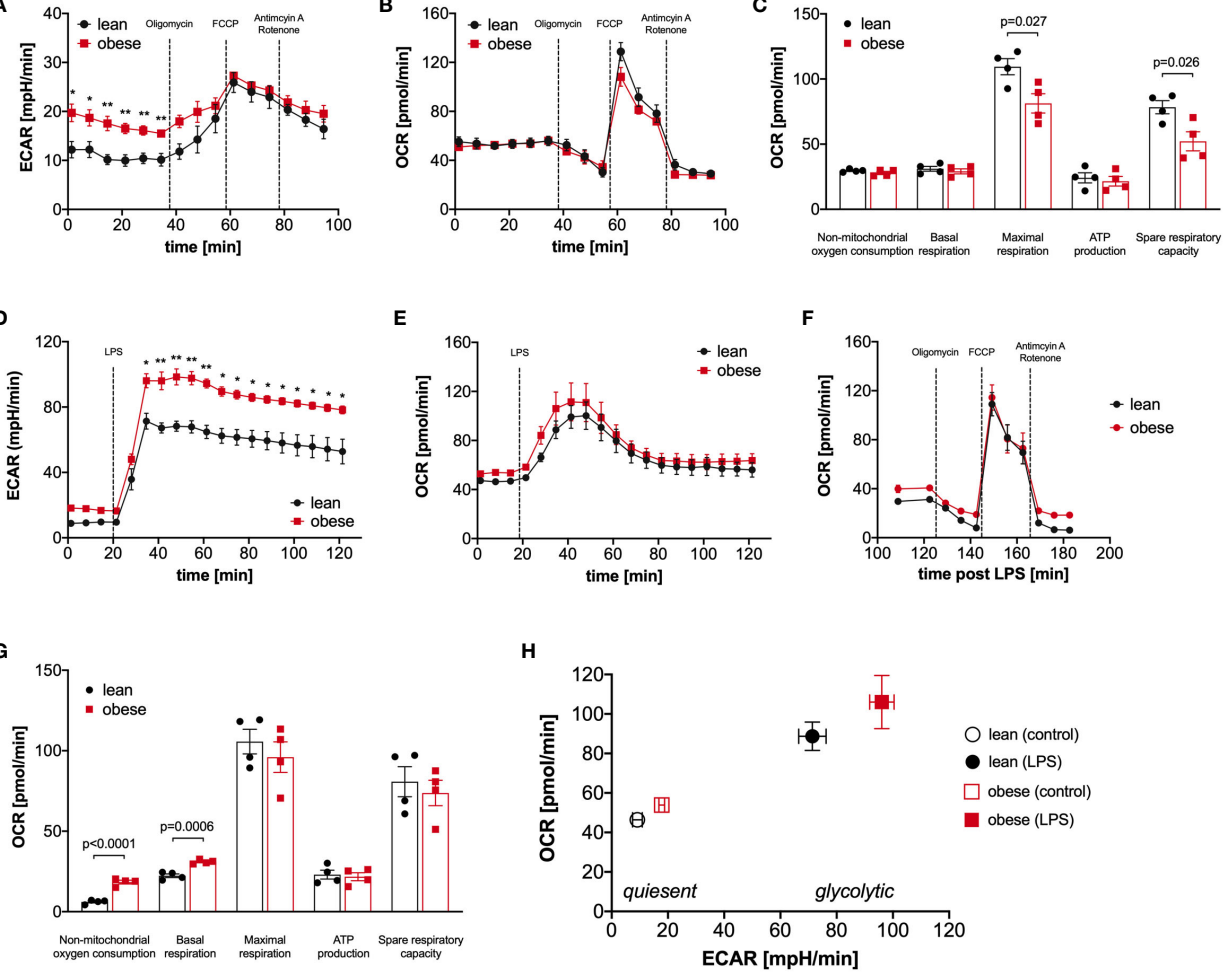
Monocytes of obese people have a hyper-glycolytic phenotype. Seahorse bioenergetics profile (mean ± SEM) demonstrating **(A)** baseline ECAR and **(B)** OCR of *ex vivo* quiescent monocytes of lean and obese people (n=4) before and after injections of oligomycin, FCCP and antimycin A/rotenone. Baseline respiration, ATP synthesis, maximal respiratory capacity, non-mitochondrial oxygen consumption, and spare respiratory capacity [**(C)** mean ± SEM]. Seahorse bioenergetics profile (mean ± SEM) demonstrating **(D)** ECAR and **(E)** OCR of monocytes of lean and obese people (n=4) before and after injections of 100 ng/ml LPS. **(F, G)** Seahorse bioenergetics profile (mean ± SEM) demonstrating OCR of LPS-stimulated monocytes of lean and obese people (n=4) before and after injections of oligomycin, FCCP and antimycin A/rotenone **(F)**. Baseline respiration, ATP synthesis, maximal respiratory capacity, non-mitochondrial oxygen consumption, and spare respiratory capacity (**(G)** mean ± SEM). **(H)** Overall metabolic profile of quiescent and LPS‐stimulated monocytes of lean and obese people (n=4). Statistical analysis was performed using *t*-test. *P < 0.05, **P < 0.01.

The proteomic profiling revealed that metabolic pathways like glycolysis and TCA cycle are significantly regulated in LPS-activated monocytes of donors with obesity compared to lean people (**Figure 2C**, **Additional File Table A3**). Therefore, we next analyzed glucose utilization and oxygen consumption in monocytes using the Seahorse-XFe96 analyzer. Quiescent, i.e. unstimulated, monocytes of people with obesity showed an increased glycolytic rate in comparison to monocytes of lean people (ECAR, extracellular acidification rate, **Figure 3A**), whereas the oxygen consumption rate (OCR) was not different (**Figure 3B**). To analyze basal respiration, maximal respiration, ATP production, spare respiratory capacity, and non-mitochondrial respiration in mitochondria, respiratory chain inhibitors and uncouplers were successively added. As shown in **Figure 3C**, monocytes of people with obesity had lower maximal respiration and spare respiratory capacity than monocytes of lean people.

Monocytes are known to undergo a switch from oxidative phosphorylation to glycolysis in response to activation, the so-called Warburg effect (25, 26). When LPS was added to the monocytes, we observed, as expected, a steep increase in the glycolytic rate (**Figure 3D**), and the already higher glycolytic rate of monocytes from people with obesity further increased in comparison to monocytes of lean people. This hyper-glycolytic phenotype was accompanied by a normal oxygen consumption rate (**Figure 3E**) and a normal mitochondrial profile (**Figure 3F**). Interestingly, the non-mitochondrial oxygen consumption was markedly increased in monocytes of people with obesity (**Figure 3G**), pointing to increased oxidative reactions not linked to energy metabolism. **Figure 3H** summarizes the metabolic switch following LPS stimulation, and whereas both monocytes from lean and people with obesity switch their metabolism to a highly energetic state, monocytes from people with obesity show a hyper-glycolytic phenotype both in the quiescent and activated state.

**Figure 4:**
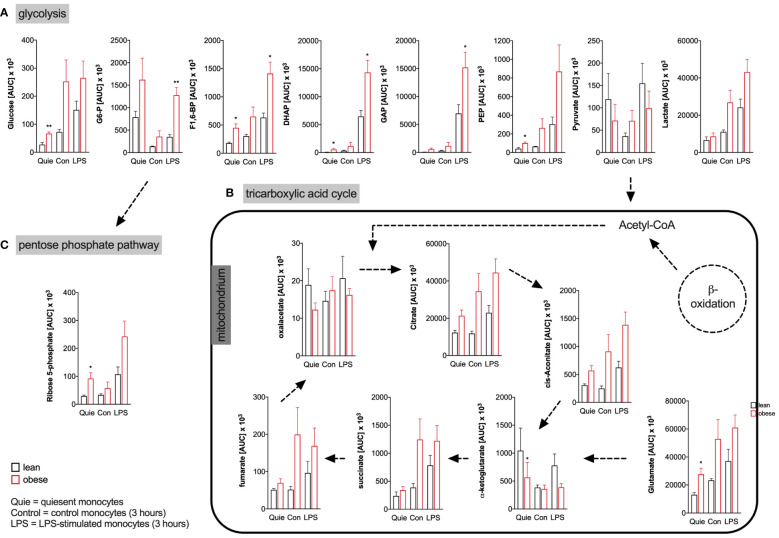
Metabolites of glycolysis, pentose phosphate pathway, and tricarboxylic acid cycle of monocytes from donors with overweight, obesity, obesity/IGT and obesity/T2D compared to monocytes from lean donors. **(A)** Intracellular glycolytic, **(B)** tricarboxylic acid cycle, and **(C)** pentose phosphate pathway metabolites were quantified from quiescent (ex vivo), 3 hour control and LPS-stimulated monocytes of lean and obese people (n=4) with methanol/chloroform and measured using LC-MS/MS. Shown are mean values of peak areas (± SEM). Statistical analysis was performed using *t*-test. *P < 0.05, **P < 0.01.

The targeted assessment of metabolites of the central carbon metabolism of quiescent and LPS-activated monocytes from lean and people with obesity revealed that intracellular glucose and most glycolysis intermediates are increased in monocytes of people with obesity (**Figure 4A**). Analysis of TCA metabolites showed that quiescent and LPS-activated monocytes from lean and people with obesity did not differ in TCA metabolite concentrations (**Figure 4B**). The pentose phosphate pathway (PPP) intermediate ribose-5-phosphate was found to be increased in quiescent monocytes of people with obesity compared to lean people (**Figure 4C**).

**Figure 5:**
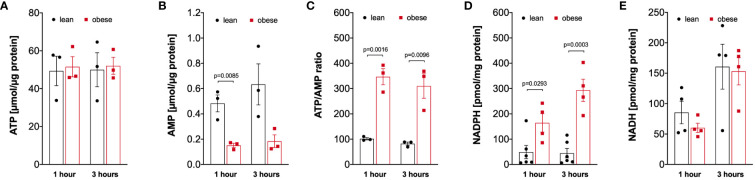
Altered intracellular nucleotides in monocytes of people with obesity. **(A)** Intracellular ATP (n=3), **(B)** AMP (n=3), **(D)** NADPH (lean n=6, obese n=4), and **(E)** NADH (n=4) nucleotides were extracted from 1-hour and 3-hour LPS-stimulated monocytes of lean and obese people, **(C)** shows the calculated ATP/AMP ratio. Statistical analysis was performed using *t*-test.

Analysis of the energy status of LPS-activated monocytes revealed that monocytes of lean and people with obesity have comparable amounts of intracellular ATP (**Figure 5A**), whereas the intracellular AMP concentration is decreased in monocytes from people with obesity (**Figure 5B**), resulting in a markedly increased ATP/AMP ratio in monocytes from people with obesity (**Figure 5C**). In addition, the concentration of NADPH, which is mainly produced in the pentose-phosphate-pathway, was increased in LPS-stimulated monocytes of people with obesity compared to lean people (**Figure 5D**). In contrast, NADH was found in equal concentrations in monocytes from both lean people and people with obesity (**Figure 5E**).

### Hyper-glycolytic monocytes show a dysregulated IL-8 response and produce elevated levels of reactive oxygen species

Obesity is associated with low-grade inflammation, a dysfunctional immune response, an increased susceptibility to infections, and a more severe disease course (9, 27). This prompted us to analyze the cytokine response, the ability to phagocytose, and the oxidative burst in monocytes of people with obesity, as these are hallmarks of the innate immune system response to bacteria and bacterial products.

**Figure 6:**
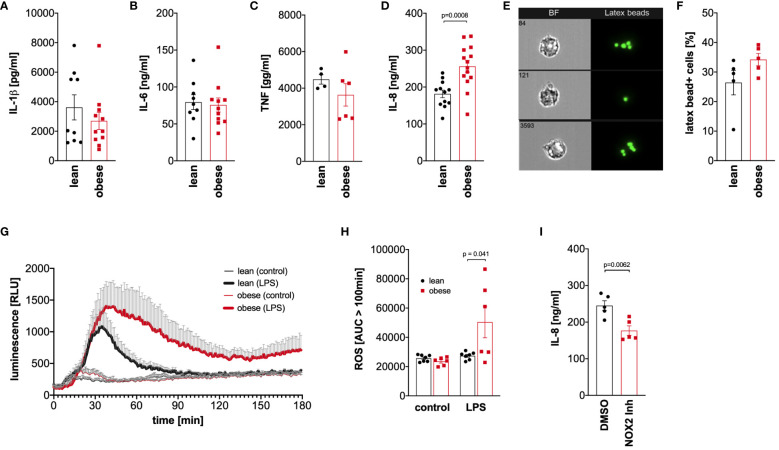
Dysregulated cytokine and oxidative response to LPS in monocytes of people with obesity. **(A-C)** Monocytes were stimulated with 100 ng/ml LPS for 16 hours (IL-1ß, IL-6, IL-8) or 4 hours (TNF), and the cytokine concentrations were determined in the supernatant by ELISA. Shown are mean values (lean n=9, obese n=11 (IL-1ß, IL-6); lean n=4, obese n=6 (TNF); lean n=12, obese n=14 (IL-8)) ± SEM. Statistical analysis was performed using *t*-test. **(D, E)** Monocytes of lean and obese people were incubated in the presence of 100 ng/ml LPS and 1 µm latex beads for 16 hours and phagocytosis was quantified by ImageStream cytometry. **(D)** Shown are representative images and **(E)** mean values (n=5) ± SEM. Statistical analysis was performed using Mann–Whitney U‐test. **(F, G)** Monocytes were stimulated with 100 ng/ml LPS or left untreated and the oxidative burst monocytes from lean (n=7) and obese people (n=6) was measured by luminescence increase of oxidized luminol. **(F)** Shown is the mean luminescence ± SEM measured for 180 minutes and **(G)** calculated area under the curve (AUC) for 100 minutes to 180 minutes. Statistical analysis was performed using *t*-test. **(H)** Monocytes of obese people (n=5) were stimulated with 100 ng/ml LPS for 16 hours in the presence of GSK2795039 (GSK) or the vehicle control (DMSO), and **(I)** released IL-8 was determined in the supernatant by ELISA. Shown are mean values ± SEM. Statistical analysis was performed using *t*-test.

Monocytes of lean people and people with obesity were activated with LPS for 4 hours to analyze TNF release or 16 hours to analyze IL-1ß, IL-6, and IL-8 release, and cytokine concentrations were analyzed in the supernatant. Hyper-glycolytic monocytes of people with obesity showed a normal IL-1ß (**Figure 6A**), IL-6 (**Figure 6B**), and TNF response (**Figure 6C**), whereas the IL-8 secretion in response to LPS was increased compared to monocytes of lean people (**Figure 6D**).

To analyze phagocytosis, monocytes of lean people and people with obesity were incubated with latex beads in the presence of bacterial LPS, and phagocytosis was quantified by ImageStream cytometry (**Figure 6E**). As shown in **Figure 6F**, monocytes of both lean people and people with obesity phagocytosed equal amounts of beads after activation with bacterial LPS.

Monocytes kill phagocytosed pathogens by the generation of reactive oxygen species in a process known as oxidative burst. This process is tightly regulated to avoid excess cell and tissue damage (28). Monocytes of lean people and people with obesity were activated with LPS, and the generation of reactive oxygen species was monitored using luminol-based chemiluminescence. As shown in **Figure 6G**, the LPS-induced oxidative burst in monocytes of lean people was limited to approximately 100 minutes, whereas monocytes of people with obesity showed a prolonged response. The amount of produced reactive oxygen species after this time point was significantly increased in monocytes of people with obesity compared to lean people (**Figure 6H**).

To test whether the increased production of reactive oxygen species in response to LPS is connected to the increased IL-8 secretion, the NOX2 inhibitor GSK2795039 was used to inhibit the NADPH oxidase 2. As shown in **Figure 6I**, inhibition of NADPH oxidase 2 led to a decreased LPS-induced IL-8 secretion in monocytes of people with obesity.

### Monocytes from people with obesity exhibit a higher number of lipid droplets compared to lean controls

**Figure 7:**
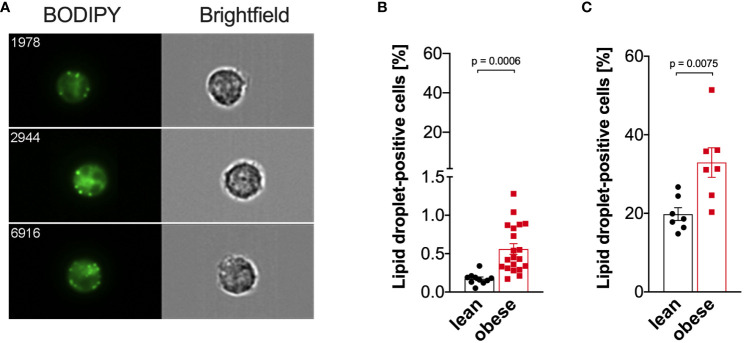
Increased lipid droplet formation in monocytes of people with obesity. Lipid droplets were stained with BODIPY and quantified by ImageStream cytometry. **(A)** Representative images of lipid droplet-positive monocytes. **(B)** Lipid droplets in freshly isolated monocytes of lean people (n=10) and people with obesity (n=20). **(C)** Lipid droplets in LPS-stimulated (16 hours) monocytes of lean people (n=7) and people with obesity (n=7). Shown are mean values ± SEM. Statistical analysis was performed using *t*-test.

As cellular metabolism was altered in monocytes from people with obesity compared to lean controls, we hypothesized that lipid droplet content differs between those two groups. Thus, the percentage of monocytes with lipid droplets was quantified using imaging flow cytometry (**Figure 7A**). Compared to freshly isolated monocytes from lean donors, the percentage of lipid droplet-positive monocytes from donors with obesity was increased (**Figure 7B**), and that was even more dramatic when cells were stimulated with LPS (**Figure 7C**).

## Discussion

Adipose tissue macrophages (ATMs) significantly contribute to the pathogenesis of obesity. Since peripheral monocytes can differentiate to ATMs and obesity is associated with enhanced myelopoiesis and monocytosis (2, 18, 20), we aimed to assess whether peripheral blood monocytes in obesity are already metabolically and inflammatory imprinted prior to fat tissue infiltration. We and others have reported previously that the absolute monocyte number in the peripheral blood is increased in people with obesity (18, 19, 29). We also observed a dysregulation of monocyte subsets in obesity with an expansion of classical and intermediate monocytes (18), which led us to focus our analysis on classical monocytes, as these are the cells that will subsequently infiltrate the fat tissue (14, 30). Through LC-MS/MS-based global proteomics, a comprehensive analysis revealed distinct molecular patterns of classical monocytes *ex vivo* in individuals with varying degrees of obesity and associated comorbidities, compared to lean donors. Notably, monocytes from people with obesity exhibited the most differences compared to lean donors, with only one overlap in the regulated proteins across the groups, namely PITH domain containing-protein 1 (PITHD1). To date, the role of PITHD1 in monocytes or macrophages has not been investigated. Thus, the role of upregulated PITHD1 in monocytes from individuals with obesity needs to be clarified in further studies. In megakaryocytes PITHD1 was shown to upregulate the expression of the transcription factor RUNX1 (23). In neutrophils, RUNX1 is involved in regulating TNF upon TLR4-stimulation and a loss in RUNX1 resulted in hyperresponsiveness to LPS (31). Another study found, that RUNX1 is downregulated after TLR4 activation by LPS in macrophages and knock-down of RUNX1 induced IL-1b and IL-6, while TNF was not affected (32). Unfortunately, RUNX1 was not detected in our dataset, due to the usually low abundance of transcription factors hampering their identification and quantification by LC-MS/MS. Alternative methods like Western blot or flow cytometry or qPCR might allow quantification of RUNX1 in monocytes. Nevertheless, we also observed changes in cytokine release in monocytes from donors with obesity after LPS i.e. TLR4 stimulation, whereby TNF, IL-6, and IL-1 were not affected, while IL-8 was increased. In acute myeloid leukemia, RUNX1 was shown to be involved in IL-8 regulation. It would be interesting to further investigate the biological relevance of the PITHD1-RUNX1 axis in monocytes.

Besides, we observed an activation of pathways relevant for cellular migration, especially in donors with overweight, indicating a potentially higher capacity to infiltrate tissues including adipose tissue, which may be further explored in future studies. Changes in phagocytosis, endocytosis and cellular metabolism were also detected that suggest a dysregulated state compared to lean donors, confirming and extending the results of our previous study (18).

After LPS-stimulation, monocytes from people with obesity showed impaired metabolic and immune responses compared to lean controls. Significant changes in the protein profiles of LPS-activated monocytes from donors with obesity and associated comorbidities included enzymes involved in cell metabolism such as aldolase A, fumarase, phosphoglycerate mutase 1 and isocitrate dehydrogenase 1. In addition, upregulation of protein disulfide isomerase family A member 4 (PDIA4), an endoplasmic stress response protein, known for its role in modulating the immune response and insulin resistance in muscle cells (24), suggests an intricate molecular adaptation of monocytes from individuals with obesity that might be involved in developing comorbidities.

In support of this finding, the pathway analysis of LPS-activated monocytes revealed remarkable changes in metabolic pathways, including glycolysis, gluconeogenesis and the TCA cycle, that were even more pronounced in monocytes from subjects with obesity compared to lean donors and went beyond the well-characterized metabolic reprogramming associated with monocyte and macrophage activation (33). It is well known that ATMs reprogram their cellular energy metabolism towards increased glycolysis but also towards increased oxidative phosphorylation with glycolysis being the main contributor to the pro-inflammatory re-programming of ATMs (34, 35). Not much is known about the cellular energy metabolism of circulating monocytes from people with obesity, however, a very recent publication suggests that the balance between glycolysis and oxidative phosphorylation is related to obesity-associated insulin resistance progression (36). It would be interesting to address in further studies if the observed glycolytic phenotype of monocytes from people with obesity leads to epigenetic reprogramming of monocyte-derived macrophages as observed in trained immunity (37), and if those macrophages resemble ATMs. In addition, it has been proposed that the inflamed adipose tissue signals to the bone marrow to produce more monocytes, further fueling inflammation and associated comorbidities (2, 20, 38). Apart from the proteomic analysis, we did not independently analyze obesity with IGT or T2D or obesity without comorbidities in the functional assays, however, this will be addressed in further studies.

More specifically, our study showed that LPS-induced activation of monocytes from obese individuals led to increased metabolic reprogramming towards glycolysis. This hyper-glycolytic phenotype, in conjunction with dysregulated cytokine responses and increased oxidative stress, may contribute to the increased susceptibility to infection observed in obese individuals. This goes in line with the observation that glycolytic monocytes have an increased viral load when challenged with SARS-CoV2, respiratory syncytial virus, or influenza A H1N1 virus (39). Glycolytic monocytes are observed in both infectious settings and inflammatory diseases. Glycolysis fuels the inflammatory program of monocytes in rheumatoid arthritis (40), in patients with atherosclerotic coronary artery disease (41), in myocardial infarction (42), in Chagas disease (43), and in malaria (44).

In addition, the increased accumulation of lipid droplets in obese monocytes emphasizes the altered cellular metabolism associated with obesity. Besides, it was previously shown that lipid droplet formation in monocytes results from increased circulating free fatty acids and was suggested as a potential biomarker of atherosclerotic cardiovascular diseases (45), a classical comorbidity of obesity. In general, lipid droplets fuel metabolic processes and membrane biogenesis after mobilization by lipolysis or lipophagy, protect against endoplasmatic reticulum stress and mitochondrial damage during autophagy (46). Lipid droplet accumulation in macrophages leads to foam cell formation (47) but little is known about the functional consequences of lipid droplet accumulation in monocytes. However, monocytes enter the arterial wall during atherosclerotic plaque formation (48, 49) and one might speculate that lipid droplet loaded monocytes might accelerate this process by differentiating into lesional macrophages and later foam cells (50) but also by lipid transport into the lesions (51). The same might be true for the differentiation of adipose tissue foam cells and the lipid transport into the adipose tissue (52).

We observed an increased IL-8 secretion by monocytes from people with obesity. Circulating IL-8 concentrations are increased in people with obesity compared to lean individuals (53–55), which is considered to contribute to the chronic low-grade inflammation leading to the development of type 2 diabetes. Accordingly, circulating IL-8 levels have been shown to correlate with insulin resistance, whereas weight loss decreased circulating IL-8 levels but had no effect on insulin sensitivity (53). Adipocytes from visceral adipose tissue are considered the main source of elevated circulating IL-8 levels (56), but our results point to circulating monocytes as an additional source of elevated IL-8 levels in donors with obesity, potentially linking obesity and its associated complications. IL-1b, IL-6, and TNF were not significantly regulated in monocytes of people with obesity at the investigated time points but it would be interesting to take a closer look pro-inflammatory potential of the lipid droplet positive monocytes in further studies or in a time series-dependent analysis. Dias et al. reported that inhibition of lipid droplet formation in SARS-CoV2-infected monocytes also lowered the secretion of pro-inflammatory mediators (39).

Our finding on the increased production of reactive oxygen species in monocytes of people with obesity goes in line with Degasperi et al. (57). The observed effect in monocytes of obese people might also be the result of the increased number of monocytic myeloid-derived suppressor cells (M-MDSC) present in the classical monocyte subpopulation in obese people. We have reported previously, that M-MDSCs represent 12% of classical monocytes in obese people compared to 5% in lean people (18). MDSCs are known to produce reactive oxygen species (58) and metabolic reprogramming towards glycolysis protects against ROS-mediated apoptosis (59). Further studies are needed to evaluate the role of M-MDSCs in obese people, since M-MDSCs facilitate tumor growth by various mechanisms and obesity is associated with an increased risk to develop various cancers (60). *Ex vivo* functional analysis of M-MDSCs is challenging, however, due to the low cell number in the peripheral blood.

The increased IL-8 response in monocytes from people with obesity was found to be partially depended on the NADPH oxidase as a Nox2 inhibitor led to a decreased IL-8 secretion. Hidalgo et al. observed a Nox2-dependent IL-8 secretion in neutrophils (61) and Hu et al. an decreased cytokine response in central nervous system monocytes from Nox2-deficient mice (62). However, Nox2-deficient THP-1 macrophages as a model of chronic granulomatous disease showed increased inflammasome activation and cytokine secretion (63), whereas NADPH-derived oxygen radicals played no role in dendritic cell cytokine production (64).

Limitations of this study are the absence of monocytes from female overweight donors in the proteome analysis, the difference in the median age of lean donors and people with obesity, and as already discussed, the joint analysis of monocytes from people with obesity independent of their comorbidities. Notably, we were only able to include male overweight donors within the proteome analysis. Sex-specific aspects of the proteome of monocytes from donors with overweight and obesity may be addressed in further studies. Regarding the effects on the glycolytic rate, no influence of sex on glycolytic rate of monocytes activated by bacterial stimuli was previously observed (65). We also cannot completely rule out the influence of age on our results. While the donors used in the proteomic analysis were age-matched, in the subsequent experiments we also included more young donors in the lean group which resulted in a difference in median age between the lean group and the other groups. However, the age of the donors (median age from 33 to 49 years) in all groups is not considered “old” in the context of immunosenescence, where a dysregulated immune response is observed in people older than 65 or 70 years (66). In addition to the dysregulated immune response in aging, the metabolism is also influenced by aging. Pence et al. reported a mitochondrial dysfunction in monocytes from individuals 60-80 years of age compared to young individuals (18-35 years) (67), and additional investigation is needed to delineate the mechanisms behind age-specific effects in older people with obesity.

Taken together, the pathway analysis, the oxidative burst assay as well as cytokine release data confirmed a dysregulated LPS-response in monocytes from donors with obesity, which potentially increases the risk for infections. Nevertheless, further research is needed to elucidate the mechanisms underlying the observed changes in monocyte function in obesity. Longitudinal studies investigating the dynamic changes in metabolism and immune cell function during the progression of obesity and associated comorbidities could provide valuable insights into potential therapeutic targets.

## Methods

### Study participants

A total of 151 participants, 52 lean (18 male, 34 female), 6 overweight (6 male), and 93 obese individuals (27 male, 66 female), were recruited from the Integrated Research and Treatment Center Adiposity Diseases of the Medical Faculty of Leipzig University and the University Hospital Leipzig. All study participants were older than 18 years of age. The experimental design of the clinical study has been approved by the ethics committee of the University of Leipzig (Approval number 017-12ek). Informed and written consent was obtained from all individuals before the enrollment to the study.

The classification of normal, overweight, and obese was done according to the definition of the World Health Organization (WHO) based on the body mass index (BMI; body weight in kilograms, divided by height in meters squared; normal BMI 18.5–24.9; overweight BMI 25.0–29.9; obese BMI above 30). A 75 g, 2 h, oral glucose tolerance test (OGTT) was performed with obese people according to the WHO criteria. Obese individuals were categorized according to the glycemic status into groups with impaired glucose tolerance (IGT; OGTT > 140 mg/dl) and with normal glucose tolerance (OGTT < 140 mg/dl). Patients with T2D, were classified according to the criteria of the American Diabetes Association (HbA1c levels > 48 mmol/mol and/or OGTT > 200 mg/dl). Characteristics of study participants are shown in **Supplementary Table 1**. In the initial proteomics analysis, all groups (lean, overweight, obese, obese with IGT, and obese with T2D) were analyzed. In all the subsequent experiments, monocytes from lean donors were compared with monocytes from people with obesity independent of the presence or absence of comorbidities.

### Monocyte isolation and culture

Peripheral blood mononuclear cells (PBMCs) were isolated by density gradient centrifugation using Ficoll-Paque (GE healthcare). PBMCs were washed with cold PBS containing 0.3 mM ETDA and monocytes were isolated by negative selection using the human classical monocyte isolation kit (Miltenyi Biotech) according to the manufacturer’s instructions. Monocyte purity was found to be >95%. A representative CD14/CD16 staining (analyzed by flow cytometry) is shown in **Supplementary Figure 3**.

Monocytes were cultured in modified RPMI1640 cell culture medium (Gibco, Life Technologies) supplemented with 5 mM glucose and 10% fetal calf serum (FCS, Gibco, Life Technologies) and either left unstimulated or stimulated with 100 ng/ml LPS (Ultrapure LPS from E.coli 0111:B4, Invivogen).

### Proteomics

For proteomic analysis, 1x 10^6 monocytes per replicate and donor were cultured in modified RPMI1640 cell culture medium (Gibco, Life Technologies) supplemented with 5 mM glucose and 10% fetal calf serum (FCS, Gibco, Life Technologies) and either left unstimulated or stimulated with 100 ng/ml LPS (Ultrapure LPS from E.coli 0111:B4, Invivogen). For the analysis of the ex vivo/quiescent samples, 1x 10^6^ monocytes were lysed directly after purification. The protein lysates were prepared using RIPA lysis buffer as described previously (68). Briefly, 1 ml lysis buffer was composed of 500 µl 2x RIPA buffer (2% Triton X100, 300 mM NaCl, 100 mM Tris-HCl pH 7.4, 1% Sodium deoxycholate, 0.2% SDS), 100 µl Complete™ Protease Inhibitor Cocktail (Merck), and 400 µl sterile distilled water. Cells were washed twice with cold PBS, resuspended in lysis buffer, and incubated for 30 min on ice. Supernatants (lysates) containing proteins were collected after centrifugation at 10,000 × *g* for 15 min at 4 °C. The protein concentration in the lysates was determined using DC™ Protein Assay (Bio Rad).

20 µg protein per sample were prepared for untargeted proteomics. For this purpose, a paramagnetic bead approach was applied, which leads to improved sample quality and allows the offline fractionation of samples (69, 70). Furthermore, this paramagnetic bead approach can be combined with tandem mass tag (TMT) labeling. The applied workflow has been specified elsewhere (71). In brief, the protein samples were reduced with TCEP (Tris(2-carboxyethyl)phosphine hydrochloride, Sigma-Aldrich, USA) for 1 h at 55°C and alkylated with iodoacetamide (Merck KGaA, Germany) for 30 min in the dark at room temperature. Acetonitrile (ACN) was added to the samples to enable protein binding to SpeedBeads™ magnetic carboxylate modified particles (SP3 beads, Sigma Aldrich, Germany). Importantly, the samples were not acidified before application to the SP3 beads. Once the proteins were bound to the beads, they were rinsed twice with 70% (v/v) ethanol and once with 100% (v/v) ACN. Afterwards, proteins were digested overnight using trypsin (enzyme:protein ratio 1:50, Promega, USA). Next day, TMT labeling (TMT-11-plex, Thermo Scientific, USA) was conducted with 0.08 mg label per sample for 1 h at room temperature. Notably, this was less than recommended in the manufacturer’s instructions but has been shown to reveal sufficient labeling before (72) and led to more than 99% labeled peptides here (**Additional File Table A1**). The addition of hydroxylamine quenched labeling, labeled samples were combined (**Additional File Table A1**), and ACN was added to enable peptide binding to the SP3 beads. The bound peptides were rinsed with 100% (v/v) ACN and eluted in two steps, first with 87% (v/v) ACN in 10 mM ammonium formate (pH 10) (Agilent Technologies, USA), and then with 2% (v/v) dimethyl sulfoxide (DMSO, Sigma Aldrich, Germany). These two fractions were evaporated to dryness and reconstituted in 0.1% (v/v) formic acid (FA).

The labeled samples were analyzed on a nano-UPLC system (Ultimate 3000, Dionex, USA) with trapping column (flow rate 5 µl/min, Acclaim PepMap 100 C18, 3 µm, nanoViper, 75 µm × 5 cm, Thermo Fisher, Germany) and analytical column (flow rate 0.3 µl/min, Acclaim PepMap 100 C18, 3 µm, nanoViper, 75 µm × 25 cm, Thermo Fisher, Germany). For peptide separation, a non-linear gradient of 150 minutes was applied. Eluted peptides were ionized using a chip-based ESI source (Nanomate, Advion, USA), coupled to the mass spectrometer (QExactive HF, Thermo Scientific, USA) using the previously specified parameters for TMT samples (71), with the exception that not the top 10 but the top 15 most abundant precursor ions were isolated and fragmented.

MS raw data were processed using ProteomeDiscoverer 2.4. The database search was performed against the UniprotKB reference proteome of *Homo sapiens* (3 May 2020), selecting trypsin as cleavage reagent and allowing up to two missed cleavages. Oxidation of methionine and acetylation of the protein N-terminus were defined as variable modifications, while carbamidomethylation and TMT were selected as static modifications. Protein and peptide false discovery rates (FDRs) were set to 0.01. Only proteins for which at least two peptides were identified (one needed to be unique for the protein) were kept. Reporter ion intensities were corrected using the factors provided by the manufacturer. The co-isolation threshold was set to 50%, and the reporter ion intensities were normalized using the total peptide amount.

The subsequent analysis and related visualizations were performed in R-3.6.1 with the use of the packages limma (73), plyr (74), reshape2 (75), xlsx (76), DEP (77), calibrate (78), readxl (79), qpcR (80), splitstackshape (81), tidyr (82), Tmisc (83), ggplot2 (84), circlize (85), ggsci (86), dendsort (87), and dendextend (88).

The obtained TMT reporter ion intensities were subjected to TMT mix-internal normalization, to remove potential measurement bias. To evaluate the impact of obesity and comorbidities ex vivo, data were normalized to the respective pools (pool of equal protein amounts from all ex vivo samples) measured in the same TMT mix. To evaluate the effect of LPS stimulation in obesity, the normalization was performed calculating the TMT mix-internal ratio plus LPS vs no LPS. Furthermore, data were filtered for those proteins identified at least in triplicate, log2-transformed and variance-stabilized. Average fold changes (FCs) and p-values were calculated against samples from lean donors (**Additional File Table A3**). To identify significantly altered proteins, the Student’s t-test was used, and proteins were considered significantly changed with p-value ≤ 0.05.

To unravel affected pathways, significantly altered proteins were subjected to enrichment analyses using the Ingenuity Pathway Analysis (IPA, Qiagen, Germany) tool (89). Thereby, immune cells and immune cell lines of the human organism were selected. Pathways were considered significantly enriched with Benjamini & Hochberg adjusted p-value ≤ 0.05 (**Additional File Table A4**).

### Seahorse assay

Glycolytic and mitochondrial activity of monocytes was measured using a Seahorse extracellular flux 96 analyzer (Agilent). Monocytes were seeded at a density of 4 × 10^5^ cells/100 µl RPMI1640 without FCS and adhered for 45 minutes at cell culture conditions. After two washing steps, XF RPMI 1640 pH 7.4 (Agilent) supplemented with 5% FCS, 2 mM glutamine, and 5 mM glucose added. Extracellular acidification rate (ECAR) and oxygen consumption rate (OCR) were determined at 37°C. To analyze oxidative phosphorylation following inhibitors were injected: oligomycin (1 µM; Cayman Chemicals), FCCP (2 µM; Cayman Chemicals), antimycin A (1 µM; Sigma-Aldrich), and Rotenone (1 µM; Sigma-Aldrich). Every condition was run at least in quadruplicates and a mean was calculated in the analysis.

### Measurement of intracellular polar metabolites using targeted MRM-MS/MS

Monocytes were seeded at a density of 1 × 10^6^ cells/500 µl in 24-well plates and stimulated with 100 ng/ml LPS or left untreated for 3 hours. Cells were washed several times and 500 µl cold acetonitrile wadded and incubated for 5 minutes at -20°C. Subsequently, 500 µl phenylhydrazine (50 µM) was added and incubated for 60 minutes at -20°C. The mix was transferred to a 2 ml tube and centrifuged at 14000 rpm, 4°C for 10 minutes. The supernatants were dried using a vacuum concentrator, and the samples were stored at -80°C until the analysis.

Samples were resuspended in 100 µl Milli-Q water and measured on a QTRAP 6500+^®^ system (Sciex, Framingham, MA, USA) coupled on-line with an Agilent 1290 II infinity UPLC system (Agilent Technologies Inc., Santa Clara, CA, USA). Chromatographic separation was achieved with a XSelect HSS T3 XP column (2.1 × 150 mm, 2.5 µm, 100 Å; Waters, Milford, MA, USA) equipped with a matching precolumn. Mobile phase A and mobile phase B were 10 mM tributylamine, 10 mM acetic acid, 5% methanol and 2% 2-propanol (pH 7.1) in water and 100% 2-propanol, respectively. Metabolites were eluted with the following non-linear gradient: 0-15.5 min 0.4 mL/min, 15.5-16.5 min 0.4-0.15 mL/min, 16.5-23 min 0.15 mL/min, 23-27 min 0.15-0.4 mL/min, 27-33 min 0.4 mL/min. The autosampler was kept at 5°C and the column oven was set to 40°C. Identification and relative quantification were based on specific MRM transitions for each metabolite measured in negative mode electrospray ionization. Data acquisition and analysis were performed using the Analyst^®^ software.

### Intracellular nucleotide determination

Monocytes were seeded at a density of 3 × 10^5^ cells/200 µl in 96-well plates and stimulated with 100 ng/ml LPS or left untreated for one or three hours. ATP concentrations in the lysate were determined with the ATP Determination Kit (Thermo Fisher), AMP was measured with the AMP-Glo Assay (Promega), NADH was measured with the PicoProbe NADH Fluorometric Assay Kit (BioVision), and NADPH was measured with the PicoProbe NADPH Fluorometric Assay Kit (BioVision) following the instructions. Resulting concentrations were normalized to protein concentrations of the lysate determined with DC-Protein Assay (BioRad).

### Cytokine measurements

Monocytes were seeded at a density of 3 × 10^5^ cells/200 µl in 96-well plates and stimulated with 100 ng/ml LPS for 4 hours (TNF) or 16 hours (IL-1ß, IL-6, IL-8). Human IL-1ß, IL-6, TNF, and IL-8 in cell supernatants were detected using ELISA according to the manufacturer’s instructions (OptEIA, BD Biosciences).

### Phagocytosis assay

Phagocytic activity of monocytes was determined with 1 µm amine-modified polystyrene, fluorescent yellow-green latex beads (Sigma-Aldrich). Monocytes were seeded at a density of 6 × 10^5^ cells/400 µl in an agarose-coated 48-well plate and stimulated with 100 ng/ml LPS and 15 µl of latex beads (0.008%) for 16 hours. The fluorescence of monocytes was determined using the Amnis^®^ ImageStreamX Mark II Imaging Flow Cytometer (INSPIRE for the ISX mkII Version 200.1.388.0). Uptake of latex beads was quantified using Amins IDEAS version 6.2 software.

### Measurement of the oxidative burst

Monocytes were seeded at a density of 2 × 10^5^ cells/300 µl in phenol red free RPMI 1640 supplemented with 10% FCS and stimulated with 100 ng/ml LPS or left untreated. Luminescence from oxidized luminol (140 µM, Cayman Chemicals) was measured with a plate reader (FluoStar Optima, BMG Labtech) over 180 minutes.

### Lipid droplets

1 × 10^6^ freshly isolated monocytes or LPS-activated monocytes (100 ng/ml LPS for 16 hours) were stained with BODIPY 493/503 for 20 minutes at room temperature. Cells were washed and immediately imaged with the Amnis^®^ ImageStreamX Mark II Imaging Flow Cytometer (INSPIRE for the ISX mkII Version 200.1.388.0). Lipid droplet-positive cells were quantified using Amins IDEAS version 6.2 software.

### Graphs and statistics

Graphs and statistics were prepared with GraphPad Prism 8.4.3. Bar charts represent mean + s.e.m. and individual values of each experiment are represented as symbols in bars. Normal distribution of data was checked using the Shapiro-Wilk test. Statistical significance was determined accordingly using the two-tailed non-parametric, unpaired Mann-Whitney U tests or *t* test as appropriate, confidence interval of 95%.

## Data availability statement

The datasets presented in this study can be found in online repositories. The mass spectrometry proteomics data have been deposited to the ProteomeXchange Consortium via the PRIDE (90) partner repository with the dataset identifier PXD045931 and 10.6019/PXD045931.

## Ethics statement

The studies involving humans were approved by Ethics committee of the University of Leipzig, Leipzig, Germany. The studies were conducted in accordance with the local legislation (Approval number 017-12ek) and institutional requirements. The participants provided their written informed consent to participate in this study.

## Author contributions

VR: Formal analysis, Investigation, Writing – review & editing. IK: Formal analysis, Investigation, Writing – review & editing. JB: Investigation, Writing – review & editing. MvB: Resources, Writing – review & editing. BE: Methodology, Writing – review & editing. UR-K: Methodology, Writing – review & editing. MB: Resources, Writing – review & editing. UW: Resources, Writing – review & editing. KS: Formal analysis, Visualization, Writing – original draft. MR: Conceptualization, Formal analysis, Visualization, Writing – original draft.

## References

[1] GBD 2015 Obesity Collaborators Afshin A Forouzanfar MH Reitsma MB Sur P Estep K. Health effects of overweight and obesity in 195 countries over 25 years. N Engl J Med. (2017) 377:13–27. doi: 10.1056/NEJMoa1614362 28604169 PMC5477817

[2] Nagareddy PR Kraakman M Masters SL Stirzaker RA Gorman DJ Grant RW. Adipose tissue macrophages promote myelopoiesis and monocytosis in obesity. Cell Metab. (2014) 19:821–35. doi: 10.1016/j.cmet.2014.03.029 PMC4048939 24807222

[3] Weisberg SP McCann D Desai M Rosenbaum M Leibel RL Ferrante AW. Obesity is associated with macrophage accumulation in adipose tissue. J Clin Invest. (2003) 112:1796–808. doi: 10.1172/JCI200319246 PMC296995 14679176

[4] Xu H Barnes GT Yang Q Tan G Yang D Chou CJ. Chronic inflammation in fat plays a crucial role in the development of obesity-related insulin resistance. J Clin Invest. (2003) 112:1821–30. doi: 10.1172/JCI200319451 PMC296998 14679177

[5] Hunsche C Hernandez O de la Fuente M. Impaired immune response in old mice suffering from obesity and premature immunosenescence in adulthood. J Gerontol Ser A. (2016) 71:983–91. doi: 10.1093/gerona/glv082 26219848

[6] Tam BT Morais JA Santosa S. Obesity and ageing: Two sides of the same coin. Obes Rev. (2020) 21:e12991. doi: 10.1111/obr.12991 32020741

[7] Ryu S Sidorov S Ravussin E Artyomov M Iwasaki A Wang A. The matricellular protein SPARC induces inflammatory interferon-response in macrophages during aging. Immunity. (2022) 55:1609–26. doi: 10.1016/j.immuni.2022.07.007 PMC9474643 35963236

[8] Muscogiuri G Pugliese G Laudisio D Castellucci B Barrea L Savastano S. The impact of obesity on immune response to infection: Plausible mechanisms and outcomes. Obes Rev. (2021) 22:e13216. doi: 10.1111/obr.13216 33719175

[9] Pugliese G Liccardi A Graziadio C Barrea L Muscogiuri G Colao A. Obesity and infectious diseases: pathophysiology and epidemiology of a double pandemic condition. Int J Obes. (2022) 46:449–65. doi: 10.1038/s41366-021-01035-6 35058571

[10] Piernas C Patone M Astbury NM Gao M Sheikh A Khunti K. Associations of BMI with COVID-19 vaccine uptake, vaccine effectiveness, and risk of severe COVID-19 outcomes after vaccination in England: a population-based cohort study. Lancet Diabetes Endocrinol. (2022) 10:571–80. doi: 10.1016/S2213-8587(22)00158-9 PMC9246477 35780805

[11] Neidich SD Green WD Rebeles J Karlsson EA Schultz-Cherry S Noah TL. Increased risk of influenza among vaccinated adults who are obese. Int J Obes 2005. (2017) 41:1324–30. doi: 10.1038/ijo.2017.131 PMC5585026 28584297

[12] Mohammad S Aziz R Al Mahri S Malik SS Haji E Khan AH. Obesity and COVID-19: what makes obese host so vulnerable? Immun Ageing A. (2021) 18:1. doi: 10.1186/s12979-020-00212-x PMC7779330 33390183

[13] Mir IA Soni R Srivastav SK Bhavya I Dar WQ Farooq MD. Obesity as an important marker of the COVID-19 pandemic. Cureus. (2022) 14:e21403. doi: 10.7759/cureus.21403 35198310 PMC8856632

[14] Wouters K Gaens K Bijnen M Verboven K Jocken J Wetzels S. Circulating classical monocytes are associated with CD11c^+^ macrophages in human visceral adipose tissue. Sci Rep. (2017) 7:42665. doi: 10.1038/srep42665 28198418 PMC5309742

[15] Russo L Lumeng CN. Properties and functions of adipose tissue macrophages in obesity. Immunology. (2018) 155:407–17. doi: 10.1111/imm.13002 PMC6230999 30229891

[16] Liang W Qi Y Yi H Mao C Meng Q Wang H. The roles of adipose tissue macrophages in human disease. Front Immunol. (2022) 13:908749. doi: 10.3389/fimmu.2022.908749 35757707 PMC9222901

[17] Lauterbach MAR Wunderlich FT. Macrophage function in obesity-induced inflammation and insulin resistance. Pflüg Arch - Eur J Physiol. (2017) 469:385–96. doi: 10.1007/s00424-017-1955-5 PMC5362664 28233125

[18] Friedrich K Sommer M Strobel S Thrum S Blüher M Wagner U. Perturbation of the monocyte compartment in human obesity. Front Immunol. (2019) 10:1874. doi: 10.3389/fimmu.2019.01874 31440251 PMC6694869

[19] Kullo IJ Hensrud DD Allison TG. Comparison of numbers of circulating blood monocytes in men grouped by body mass index (<25, 25 to <30, > or =30). Am J Cardiol. (2002) 89(12):1441–3. 10.1016/s0002-9149(02)02366-4 12062747

[20] McDowell SAC Milette S Doré S Yu MW Sorin M Wilson L. Obesity alters monocyte developmental trajectories to enhance metastasis. J Exp Med. (2023) 220:e20220509. doi: 10.1084/jem.20220509 37166450 PMC10182775

[21] Devêvre EF Renovato-Martins M Clément K Sautès-Fridman C Cremer I Poitou C. Profiling of the three circulating monocyte subpopulations in human obesity. J Immunol. (2015) 194:3917–23. doi: 10.4049/jimmunol.1402655 25786686

[22] Krinninger P Ensenauer R Ehlers K Rauh K Stoll J Krauss-Etschmann S. Peripheral monocytes of obese women display increased chemokine receptor expression and migration capacity. J Clin Endocrinol Metab. (2014) 99:2500–9. doi: 10.1210/jc.2013-2611 24606068

[23] Lu B Sun X Chen Y Jin Q Liang Q Liu S. Novel function of PITH domain-containing 1 as an activator of internal ribosomal entry site to enhance RUNX1 expression and promote megakaryocyte differentiation. Cell Mol Life Sci CMLS. (2015) 72:821–32. doi: 10.1007/s00018-014-1704-2 PMC11113685 25134913

[24] Lee CH Chiang CF Lin FH Kuo FC Su SC Huang CL. PDIA4, a new endoplasmic reticulum stress protein, modulates insulin resistance and inflammation in skeletal muscle. Front Endocrinol. (2022) 13:1053882. doi: 10.3389/fendo.2022.1053882 PMC9816868 36619574

[25] Yang L Xie M Yang M Yu Y Zhu S Hou W. PKM2 regulates the Warburg effect and promotes HMGB1 release in sepsis. Nat Commun. (2014) 5:4436. doi: 10.1038/ncomms5436 25019241 PMC4104986

[26] Raulien N Friedrich K Strobel S Rubner S Baumann S von Bergen M. Fatty acid oxidation compensates for lipopolysaccharide-induced warburg effect in glucose-deprived monocytes. Front Immunol. (2017) 8:609. doi: 10.3389/fimmu.2017.00609 28611773 PMC5447039

[27] Thomas AL Alarcon PC Divanovic S Chougnet CA Hildeman DA Moreno-Fernandez ME. Implications of inflammatory states on dysfunctional immune responses in aging and obesity. Front Aging. (2021) 2:732414. doi: 10.3389/fragi.2021.732414 35822048 PMC9261339

[28] Leavy O. Regulating ROS. Nat Rev Immunol. (2014) 14:357–7. doi: 10.1038/nri3685 24798369

[29] Thrum S Sommer M Raulien N Gericke M Massier L Kovacs P. Macrophages in obesity are characterised by increased IL-1β response to calcium-sensing receptor signals. Int J Obes 2005. (2022) 46:1883–91. doi: 10.1038/s41366-022-01135-x PMC9492543 35931812

[30] Patel AA Zhang Y Fullerton JN Boelen L Rongvaux A Maini AA. The fate and lifespan of human monocyte subsets in steady state and systemic inflammation. J Exp Med. (2017) 214(7):1913–23. doi: 10.1084/jem.20170355 PMC5502436 28606987

[31] Bellissimo DC Chen Ch Zhu Q Bagga S Lee CT He B. Runx1 negatively regulates inflammatory cytokine production by neutrophils in response to Toll-like receptor signaling. Blood Adv. (2020) 4:1145–58. doi: 10.1182/bloodadvances.2019000785 PMC7094023 32208490

[32] Luo MC Zhou SY Feng DY Xiao J Li WY Xu CD. Runt-related transcription factor 1 (RUNX1) binds to p50 in macrophages and enhances TLR4-triggered inflammation and septic shock. J Biol Chem. (2016) 291:22011–20. doi: 10.1074/jbc.M116.715953 PMC5063984 27573239

[33] Lachmandas E Boutens L Ratter JM Hijmans A Hooiveld GJ Joosten LAB. Microbial stimulation of different Toll-like receptor signalling pathways induces diverse metabolic programmes in human monocytes. Nat Microbiol. (2016) 2:1–10. doi: 10.1038/nmicrobiol.2016.246 27991883

[34] Sharma M Boytard L Hadi T Koelwyn G Simon R Ouimet M. Enhanced glycolysis and HIF-1α activation in adipose tissue macrophages sustains local and systemic interleukin-1β production in obesity. Sci Rep. (2020) 10:5555. doi: 10.1038/s41598-020-62272-9 32221369 PMC7101445

[35] Boutens L Hooiveld GJ Dhingra S Cramer RA Netea MG Stienstra R. Unique metabolic activation of adipose tissue macrophages in obesity promotes inflammatory responses. Diabetologia. (2018) 61:942–53. doi: 10.1007/s00125-017-4526-6 PMC6448980 29333574

[36] Smeehuijzen L Gijbels A Nugteren-Boogaard JP Vrieling F Boudjadja MB Trouwborst I. Immunometabolic signatures of circulating monocytes in humans with obesity and insulin resistance. Diabetes. (2024) 73:1112–21. doi: 10.2337/db23-0970 38656918

[37] Thiem K Keating ST Netea MG Riksen NP Tack CJ van Diepen J. Hyperglycemic memory of innate immune cells promotes *in vitro* proinflammatory responses of human monocytes and murine macrophages. J Immunol. (2021) 206:807–13. doi: 10.4049/jimmunol.1901348 33431659

[38] Jaitin DA Adlung L Thaiss CA Weiner A Li B Descamps H. Lipid-associated macrophages control metabolic homeostasis in a trem2-dependent manner. Cell. (2019) 178:686–698.e14. doi: 10.1016/j.cell.2019.05.054 31257031 PMC7068689

[39] Dias SSG Soares VC Ferreira AC Sacramento CQ Fintelman-Rodrigues N Temerozo JR. Lipid droplets fuel SARS-CoV-2 replication and production of inflammatory mediators. PloS Pathog. (2020) 16:e1009127. doi: 10.1371/journal.ppat.1009127 33326472 PMC7773323

[40] McGarry T Hanlon MM Marzaioli V Cunningham CC Krishna V Murray K. Rheumatoid arthritis CD14+ monocytes display metabolic and inflammatory dysfunction, a phenotype that precedes clinical manifestation of disease. Clin Transl Immunol. (2021) 10:e1237. doi: 10.1002/cti2.1237 PMC7815439 33510894

[41] Shirai T Nazarewicz RR Wallis BB Yanes RE Watanabe R Hilhorst M. The glycolytic enzyme PKM2 bridges metabolic and inflammatory dysfunction in coronary artery disease. J Exp Med. (2016) 213:337–54. doi: 10.1084/jem.20150900 PMC4813677 26926996

[42] Mouton AJ Aitken NM Moak SP do Carmo JM da Silva AA Omoto ACM. Temporal changes in glucose metabolism reflect polarization in resident and monocyte-derived macrophages after myocardial infarction. Front Cardiovasc Med. (2023) 10:1136252. doi: 10.3389/fcvm.2023.1136252 37215542 PMC10196495

[43] Sanmarco LM Eberhardt N Bergero G Palacio LPQ Adami PM Visconti LM. Monocyte glycolysis determines CD8^+^ T cell functionality in human Chagas disease. JCI Insight. (2019) 4(18):e123490. doi: 10.1172/jci.insight.123490 31479429 PMC6795286

[44] Diniz SQ Teixeira-Carvalho A Figueiredo MM Costa PAC Rocha BC Martins-Filho OA. Plasmodium vivax infection alters mitochondrial metabolism in human monocytes. mBio. (2021) 12(4):e0124721. doi: 10.1128/mBio.01247-21 34311577 PMC8406267

[45] den Hartigh LJ Connolly-Rohrbach JE Fore S Huser TR Rutledge JC. Fatty acids from very low-density lipoprotein lipolysis products induce lipid droplet accumulation in human monocytes. J Immunol. (2010) 184:3927–36. doi: 10.4049/jimmunol.0903475 PMC2843797 20208007

[46] Olzmann JA Carvalho P. Dynamics and functions of lipid droplets. Nat Rev Mol Cell Biol. (2019) 20:137–55. doi: 10.1038/s41580-018-0085-z PMC6746329 30523332

[47] Gui Y Zheng H Cao RY. Foam cells in atherosclerosis: novel insights into its origins, consequences, and molecular mechanisms. Front Cardiovasc Med. (2022) 9:845942. doi: 10.3389/fcvm.2022.845942 35498045 PMC9043520

[48] Tacke F Alvarez D Kaplan TJ Jakubzick C Spanbroek R Llodra J. Monocyte subsets differentially employ CCR2, CCR5, and CX3CR1 to accumulate within atherosclerotic plaques. J Clin Invest. (2007) 117:185–94. doi: 10.1172/JCI28549 PMC1716202 17200718

[49] Combadière C Potteaux S Rodero M Simon T Pezard A Esposito B. Combined inhibition of CCL2, CX3CR1, and CCR5 abrogates Ly6C(hi) and Ly6C(lo) monocytosis and almost abolishes atherosclerosis in hypercholesterolemic mice. Circulation. (2008) 117:1649–57. 10.1161/CIRCULATIONAHA.107.745091 18347211

[50] Singh A Sen P. Lipid droplet: A functionally active organelle in monocyte to macrophage differentiation and its inflammatory properties. Biochim Biophys Acta BBA - Mol Cell Biol Lipids. (2021) 1866:158981. doi: 10.1016/j.bbalip.2021.158981 34119681

[51] Fernandez-Ruiz I Puchalska P Narasimhulu CA Sengupta B Parthasarathy S. Differential lipid metabolism in monocytes and macrophages: influence of cholesterol loading. J Lipid Res. (2016) 57:574–86. doi: 10.1194/jlr.M062752 PMC4808766 26839333

[52] Shapiro H Pecht T Shaco-Levy R Harman-Boehm I Kirshtein B Kuperman Y. Adipose tissue foam cells are present in human obesity. J Clin Endocrinol Metab. (2013) 98:1173–81. doi: 10.1210/jc.2012-2745 23372170

[53] Kim CS Park HS Kawada T Kim JH Lim D Hubbard NE. Circulating levels of MCP-1 and IL-8 are elevated in human obese subjects and associated with obesity-related parameters. Int J Obes. (2006) 30:1347–55. doi: 10.1038/sj.ijo.0803259 16534530

[54] Straczkowski M Dzienis-Straczkowska S Stêpieñ A Kowalska I Szelachowska M Kinalska I. Plasma interleukin-8 concentrations are increased in obese subjects and related to fat mass and tumor necrosis factor-alpha system. J Clin Endocrinol Metab. (2002) 87:4602–6. doi: 10.1210/jc.2002-020135 12364441

[55] Bruun JM Verdich C Toubro S Astrup A Richelsen B. Association between measures of insulin sensitivity and circulating levels of interleukin-8, interleukin-6 and tumor necrosis factor-alpha. Effect of weight loss in obese men. Eur J Endocrinol. (2003) 148:535–42. doi: 10.1530/eje.0.1480535 12720537

[56] Bruun JM Lihn AS Madan AK Pedersen SB Schiøtt KM Fain JN. Higher production of IL-8 in visceral vs. subcutaneous adipose tissue. Implication of nonadipose cells in adipose tissue. Am J Physiol Endocrinol Metab. (2004) 286:E8–13. 13129857 10.1152/ajpendo.00269.2003

[57] Degasperi GR Denis RGP Morari J Solon C Geloneze B Stabe C. Reactive oxygen species production is increased in the peripheral blood monocytes of obese patients. Metabolism. (2009) 58:1087–95. doi: 10.1016/j.metabol.2009.04.002 19439330

[58] Corzo CA Cotter MJ Cheng P Cheng F Kusmartsev S Sotomayor E. Mechanism regulating reactive oxygen species in tumor induced myeloid-derived suppressor cells. J Immunol Baltim Md 1950. (2009) 182:5693–701. doi: 10.4049/jimmunol.0900092 PMC2833019 19380816

[59] Jian SL Chen WW Su YC Su YW Chuang TH Hsu SC. Glycolysis regulates the expansion of myeloid-derived suppressor cells in tumor-bearing hosts through prevention of ROS-mediated apoptosis. Cell Death Dis. (2017) 8:e2779. doi: 10.1038/cddis.2017.192 28492541 PMC5520713

[60] Lauby-Secretan B Scoccianti C Loomis D Grosse Y Bianchini F Straif K. Body fatness and cancer–viewpoint of the IARC working group. N Engl J Med. (2016) 375:794–8. doi: 10.1056/NEJMsr1606602 PMC6754861 27557308

[61] Hidalgo MA Carretta MD Teuber SE Zárate C Cárcamo L Concha II. fMLP-induced IL-8 release is dependent on NADPH oxidase in human neutrophils. J Immunol Res. (2015) 2015:120348. doi: 10.1155/2015/120348 26634216 PMC4655063

[62] Hu CF Wu SP Lin GJ Shieh CC Hsu CS Chen JW. Microglial nox2 plays a key role in the pathogenesis of experimental autoimmune encephalomyelitis. Front Immunol. (2021) 12. doi: 10.3389/fimmu.2021.638381 PMC8050344 33868265

[63] Benyoucef A Marchitto L Touzot F. CRISPR gene-engineered CYBBko THP-1 cell lines highlight the crucial role of NADPH-induced reactive oxygen species for regulating inflammasome activation. J Allergy Clin Immunol. (2020) 145:1690–1693.e5. doi: 10.1016/j.jaci.2019.12.913 31954113

[64] Vulcano M Dusi S Lissandrini D Badolato R Mazzi P Riboldi E. Toll receptor-mediated regulation of NADPH oxidase in human dendritic cells. J Immunol Baltim Md 1950. (2004) 173:5749–56. doi: 10.4049/jimmunol.173.9.5749 15494527

[65] Vrieling F van Dierendonck XAMH Jaeger M Janssen AWM Hijmans A Netea MG. Glycolytic activity in human immune cells: inter-individual variation and functional implications during health and diabetes. Immunometabolism Cobham Surrey Engl. (2022) 4:e00008. doi: 10.1097/IN9.0000000000000008 PMC9624385 36337734

[66] Shaw AC Goldstein DR Montgomery RR. Age-dependent dysregulation of innate immunity. Nat Rev Immunol. (2013) 13:875–87. doi: 10.1038/nri3547 PMC4096436 24157572

[67] Pence BD Yarbro JR. Aging impairs mitochondrial respiratory capacity in classical monocytes. Exp Gerontol. (2018) 108:112–7. doi: 10.1016/j.exger.2018.04.008 29655929

[68] Murthy S Karkossa I Schmidt C Hoffmann A Hagemann T Rothe K. Danger signal extracellular calcium initiates differentiation of monocytes into SPP1/osteopontin-producing macrophages. Cell Death Dis. (2022) 13:53. doi: 10.1038/s41419-022-04507-3 35022393 PMC8755842

[69] Hughes CS Foehr S Garfield DA Furlong EE Steinmetz LM Krijgsveld J. Ultrasensitive proteome analysis using paramagnetic bead technology. Mol Syst Biol. (2014) 10:757. doi: 10.15252/msb.20145625 25358341 PMC4299378

[70] Hughes CS Moggridge S Müller T Sorensen PH Morin GB Krijgsveld J. Single-pot, solid-phase-enhanced sample preparation for proteomics experiments. Nat Protoc. (2019) 14:68–85. doi: 10.1038/s41596-018-0082-x 30464214

[71] Wang Z Karkossa I Großkopf H Rolle-Kampczyk U Hackermüller J von Bergen M. Comparison of quantitation methods in proteomics to define relevant toxicological information on AhR activation of HepG2 cells by BaP. Toxicology. (2021) 448:152652. doi: 10.1016/j.tox.2020.152652 33278487

[72] Zecha J Satpathy S Kanashova T Avanessian SC Kane MH Clauser KR. TMT labeling for the masses: A robust and cost-efficient, in-solution labeling approach. Mol Cell Proteomics MCP. (2019) 18:1468–78. doi: 10.1074/mcp.TIR119.001385 PMC6601210 30967486

[73] Wickham H. The split-apply-combine strategy for data analysis. J Stat Software. (2011) 40:1–29. doi: 10.18637/jss.v040.i01

[74] Ritchie ME Phipson B Wu D Hu Y Law CW Shi W. limma powers differential expression analyses for RNA-sequencing and microarray studies. Nucleic Acids Res. (2015) 43:e47. doi: 10.1093/nar/gkv007 25605792 PMC4402510

[75] Wickham H. Reshaping data with the reshape package. J Stat Software. (2007) 21:1–20. doi: 10.18637/jss.v021.i12

[76] Dragulescu A Arendt C. xlsx: Read, Write, Format Excel 2007 and Excel 97/2000/XP/2003 Files version 0.6.5 from CRAN. R Package Version 061 (2018). Available online at: https://rdrr.io/cran/xlsx/.

[77] Zhang X Smits AH van Tilburg GB Ovaa H Huber W Vermeulen M. Proteome-wide identification of ubiquitin interactions using UbIA-MS. Nat Protoc. (2018) 13:530–50. doi: 10.1038/nprot.2017.147 29446774

[78] Graffelman J. calibrate: Calibration of Biplot and Scatterplot Axis in calibrate: Calibration of Scatterplot and Biplot Axes (2019). Available online at: https://rdrr.io/cran/calibrate/man/calibrate.html.

[79] Wickham H Bryan J. Read Excel Files [R package readxl version 1.3.1]. Comprehensive R Archive Network (CRAN (2019). Available at: https://CRAN.R-project.org/package=readxl.

[80] Spiess A. qpcR: Modelling and Analysis of Real-Time PCR Data. Comprehensive R Archive Network (CRAN (2018). Available at: https://CRAN.R-project.org/package=qpcR.

[81] Mahto A. splitstackshape: Stack and Reshape Datasets After Splitting Concatenated Values. Comprehensive R Archive Network (CRAN (2018). Available at: https://CRAN.R-project.org/package=splitstackshape.

[82] Wickham H Henry L. tidyr: easily tidy data with “spread ()” and “gather ()” functions. R package version 0.8. 0 (2018). Available online at: https://CRAN.R-project.org/package=tidyr.

[83] Turner S. Turner Miscellaneous [R package Tmisc version 1.0.0] (2019). Available online at: https://CRAN.R-project.org/package=Tmisc.

[84] Wickham H. ggplot2: Elegant Graphics for Data Analysis. Springer-Verl N Y (2016). Available at: https://ggplot2.tidyverse.org/.

[85] Gu Z Gu L Eils R Schlesner M Brors B. circlize Implements and enhances circular visualization in R. Bioinforma Oxf Engl. (2014) 30:2811–2. doi: 10.1093/bioinformatics/btu393 24930139

[86] Xiao N. Ggsci: Scientific Journal and Sci-Fi Themed Color Palettes for ggplot2 (2018). Available online at: https://cran.r-project.org/web/packages/ggsci/vignettes/ggsci.html.

[87] Sakai R Winand R Verbeiren T Moere AV Aerts J. dendsort: modular leaf ordering methods for dendrogram representations in R. F1000Research. (2014) 3:177. doi: 10.12688/f1000research 25232468 PMC4162509

[88] Galili T. dendextend: an R package for visualizing, adjusting and comparing trees of hierarchical clustering. Bioinforma Oxf Engl. (2015) 31:3718–20. doi: 10.1093/bioinformatics/btv428 PMC4817050 26209431

[89] Krämer A Green J Pollard J Jr Tugendreich S. Causal analysis approaches in Ingenuity Pathway Analysis. Bioinformatics. (2014) 30:523–30. doi: 10.1093/bioinformatics/btt703 PMC3928520 24336805

[90] Perez-Riverol Y Bai J Bandla C García-Seisdedos D Hewapathirana S KamatChinathan S. The PRIDE database resources in 2022: a hub for mass spectrometry-based proteomics evidences. Nucleic Acids Res. (2022) 50:D543–52. doi: 10.1093/nar/gkab1038 PMC8728295 34723319

