# Supplementary material for: Dysregulated cytokine and oxidative response in hyper-glycolytic monocytes in obesity
Source: Front Immunol. 2024 Jul 10;15:1416543. doi: 10.3389/fimmu.2024.1416543 (PMC11266186; doi:10.3389/fimmu.2024.1416543)
Supplement: Supplementary file 2 [file DataSheet_1.docx]

**Supplementary Data**

| Tab.1: Characteristics of the study participants | | | | |
| --- | --- | --- | --- | --- |
|  | **n** | **BMI**  **median (range)** | **age (years)**  **median (range)** | **sex (m/f)** |
|  |  |  |  |  |
| **lean** | 52 | 22.0 (18.5-24.9) | 33.5 (23-66) | 18/34 |
| **overweight** | 6 | 27.2 (27.0-29.9) | 49.0 (31-57) | 0/6 |
| **obese** | 48 | 47.9 (30.1-74.1) | 46.5 (21-66) | 16/32 |
| **obese/IGT** | 20 | 51.7 (34.6-73.0) | 44.5 (19-70) | 4/16 |
| **obese/T2D** | 25 | 46.3 (30.4-68.0) | 48.0 (29-64) | 7/18 |
|  |  |  |  |  |
| IGT impaired glucose tolerance, T2D type 2 diabetes | | | | |

**Supplementary Figure 1: Proteins assigned to IPA’s chemotaxis.**

For each comparison ex vivo and LPS-stimulated monocytes, the proteins assigned to IPA’s chemotaxis signaling pathway were extracted. Shown are Log2(FC) compared to the respective controls (monocytes of lean donors). The direction of the regulation is shown as follows: red: upregulation, blue: downregulation, white: no direction; grey: N./A.) and asterisks indicating the level of significance: *p-value ≤ 0.05, **p-value ≤ 0.01, ***p-value ≤ 0.001.


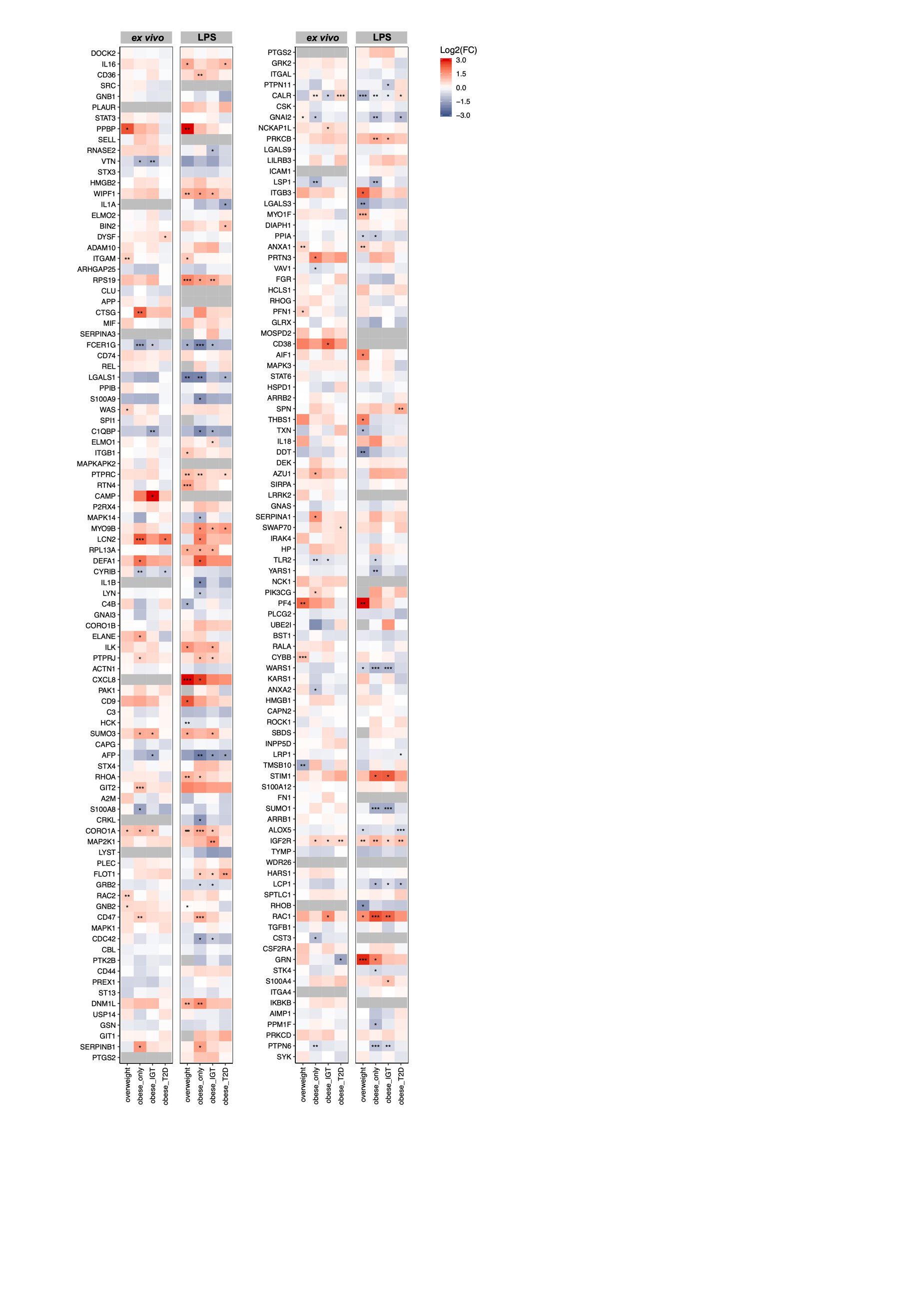

**Supplementary figure 2: Enzymes of the glycolysis and TCA cycle of LPS-stimulated monocytes from donors with obesity compared to lean donors.**

Enzymes of the IPA pathways Glycolysis I and TCA Cyle II of monocytes stimulated with 100ng/ml LPS for 3h (n=4) were extracted and depicted as Log2(FC) reflecting the direction of the regulation (red: upregulation, blue: downregulation, white: no direction; grey; no Log2(FC) available). Asterisks indicate the level of significance: * p-value ≤ 0.05, ** p-value ≤ 0.01, *** p-value ≤ 0.001.


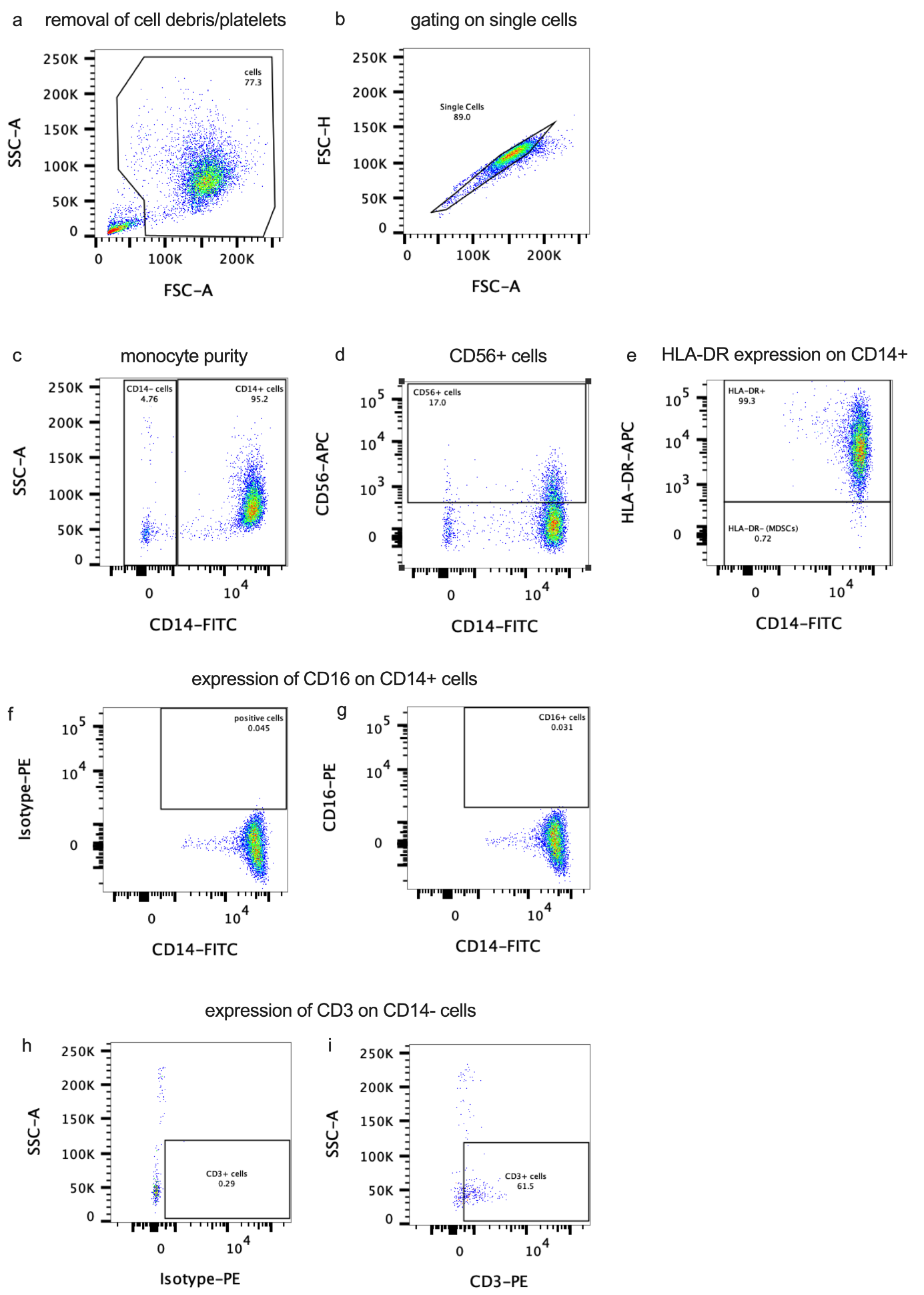


**Supplementary figure 3: Analysis of the purity of negative-separated classical monocytes**

Debris and platelets were removed from the analysis according to their FSC/SSC characteristics (a), then single cells were identified according their FCS-A/FSC-H characteristics (b), and CD14 was used to identify monocytes (c). Other markers (representative out of n=2): CD56 on all single cells (d), HLA-DR on CD14+ cells (e), CD16 and respective isotype control on CD14+ cells (f,g), and CD3 and respective isotype control on CD14- cells (h,i).
